# Supplementary material for: Pathogens Associated with Bovine Mastitis: The Experience of Bosnia and Herzegovina
Source: Vet Sci. 2024 Feb 1;11(2):63. doi: 10.3390/vetsci11020063 (PMC10891550; doi:10.3390/vetsci11020063)
Supplement: Supplementary file 1 [file vetsci-11-00063-s001.zip › vetsci-2800007-supplementary.pdf]

Table S1. Distribution of clinical and subclinical mastitis pathogens in dairy cows from different geographical regions of Bosnia and Herzegovina.

Municipality	Farm size	Clinical mastitis		Subclinical mastitis		Pathogens
No farms/ samples	No samples	No samples	No positive samples	No samples	No positive samples	
Ilijaš 2/4	large (3)			3	3	
					2	*Trueperella pyogenes*
					1	*Trueperella pyogenes/Pasteurella* spp.
	individual (1)			1	1	*Streptococcus* spp.
Ilidža 2/11	large (10)			10	7	
					4	*Staphylococcus aureus*
					1	*Pasteurella multocida*
					1	*Staphylococcus aureus/ Escherichia coli*
					1	CoNS
	individual (1)			1	1	*Streptococcus agalactiae*
Hadžići 1/2	individual (2)	2	1			*Pseudomonas aeruginosa*
**Kakanj 4/5**	individual (1)	1	1			*Staphylococcus aureus/ Escherichia coli*
	individual (1)			1	1	*Escherichia coli/Klebsiella* spp./*Enterococcus* spp./ *Candida* spp.
	individual (2)	2	2			
			1			***Mycoplasma bovis***
			1			***Mycoplasma bovis****/ Trueperella pyogenes*
	individual (1)	1				
Visoko 1/1	individual (1)	1				
**Vitez 1/38**	large [a,b]	4	2	34	13	
					3	*Streptococcus* spp.
			2			***Mycoplasma bovis***
					1	*Trueperella pyogenes*
					2	*Pasteurella multocida*
					1	CoNS
					4	*Streptococcus uberis*
					1	*Enterobacter* spp.
					1	*Prototheca zopfii*
**Doboj South 1/5**	large	5	5			***Mycoplasma bovis***
Kalesija 1/10	large [a,b]	3		7		
Gradačac 1/10	large	4	2	6	2	
			1			*Prototheca zopfii*

			1			*Streptococcus dysgalactiae* subspecies *dysgalactiae*
					1	CoNS
					1	*Streptococcus uberis*
**Goražde 1/2**	individual	2	1			***Mycoplasma bovis***
Cazin 1/11	medium [a,b]	2		9		
Bihać 1/9	medium [a,b]	9	1			*Escherichia coli*/*Streptococcus* spp.
			8			*Escherichia coli*
Čapljina 1/17	large [a,c]	1		16	3	
					2	*Enterococcus* spp.
					1	*Streptococcus* spp.
Prnjavor 1/1	small	1				
**Bijeljina 6/11**	individual	1	1			*Klebsiella* spp.
	small	2	2			***Mycoplasma bovis***
	small	1	1			***Mycoplasma bovis***/*Candida* spp.
	small	1	1			***Mycoplasma bovis***/*Escherichia coli*
		1	1			***Mycoplasma bovis***/*Streptococcus* spp.
	small	1		1	1	*Candida* spp.
	small			3		
**Pelagićevo 1/10**	medium	10	9			
			3			***Mycoplasma bovis***/ *Streptococcus agalactiae*
			2			*Streptococcus agalactiae*
			1			***Mycoplasma bovis***/*Streptococcus* spp.
			2			*Escherichia coli*
			1			*Enterobacter cloacae*
**Sokolac 3/6**	individual	1	1			***Mycoplasma bovis***
	individual [a,b]					
				1	1	CoNS
	large [a,d]	3	1	1	1	*Escherichia coli*
**Rudo 3/4**	individual	1	1			***Mycoplasma bovis***
	individual	2	2			*Streptococcus uberis*
	individual	1				
**Rogatica 1/1**	individual	1	1			***Mycoplasma bovis***/*Escherichia coli*
Han Pijesak 12/18	small			2	1	*Streptococcus agalactiae*
					1	*Staphylococcus aureus*
	individual			1	1	*Staphylococcus aureus*
	small			3		
	individual	1	1			*Staphylococcus aureus*
	small			1	1	CoNS
	small			1		
	small			4	2	CoNS
	small			1		

	small			1		
	small	1	1			*Staphylococcus aureus*
	small			1		
	small	1				
**Foča 1/1**	small	1	1			***Mycoplasma bovis***/*Escherichia coli*
Gacko	individual			1	1	*Escherichia coli*
2/2	individual			1		

[a]Farms from which BTM samples (n=8) were collected and tested: [b]farms-negative samples; [c]farm-one sample positive for *M. bovis*, one sample positive for *S. aureus*/*S. uberis*; [d]farm-one sample positive for *M. bovis*; one sample was obtained from an individual farm in Sokolac and tested negative.. The farms were classified into four herd size categories based on the number of cows: individual (≤5 cows), small (6-20 cows), medium (21- 49 cows) and large (≥50 cows). Municipalities were *M. bovis* was detected are in bold. The number of the BTM samples and the results of testing were excluded from the table.
